# Comprehensive Analysis of Myeloid Signature Genes in Head and Neck Squamous Cell Carcinoma to Predict the Prognosis and Immune Infiltration

**DOI:** 10.3389/fimmu.2021.659184

**Published:** 2021-04-29

**Authors:** Zhifeng Liu, Diekuo Zhang, Chao Liu, Guo Li, Huihong Chen, Hang Ling, Fengyu Zhang, Donghai Huang, Xingwei Wang, Yong Liu, Xin Zhang

**Affiliations:** ^1^ Department of Otolaryngology Head and Neck Surgery, Xiangya Hospital, Central South University, Changsha, China; ^2^ Otolaryngology Major Disease Research Key Laboratory of Hunan Province, Changsha, China; ^3^ Clinical Research Center for Pharyngolaryngeal Diseases and Voice Disorders in Hunan Province, Xiangya Hospital, Changsha, China; ^4^ Department of Otorhinolaryngology, The First Affiliated Hospital of University of South China, Hengyang, China; ^5^ National Clinical Research Center for Geriatric Disorders, Xiangya Hospital, Changsha, China

**Keywords:** tumor immune microenvironment, myeloid cells, head and neck squamous cell carcinoma, prognosis, immune infiltration, immunotherapy

## Abstract

Myeloid cells are a major heterogeneous cell population in the tumor immune microenvironment (TIME). Imbalance of myeloid response remains a major obstacle to a favorable prognosis and successful immune therapy. Therefore, we aimed to construct a risk model to evaluate the myeloid contexture, which may facilitate the prediction of prognosis and immune infiltration in patients with head and neck squamous cell carcinoma (HNSCC). In our study, six myeloid signature genes (including *CCL13*, *CCR7*, *CD276*, *IL1B*, *LYVE1* and *VEGFC*) analyzed from 52 differentially expressed myeloid signature genes were finally pooled to establish a prognostic risk model, termed as myeloid gene score (MGS) in a training cohort and validated in a test cohort and an independent external cohort. Furthermore, based on the MGS subgroups, we were able to effectively identify patients with a poor prognosis, aggressive clinical parameters, immune cell infiltration status and immunotherapy response. Thus, MGS may serve as an effective prognostic signature and predictive indicator for immunotherapy response in patients with HNSCC.

## Introduction

The tumor immune microenvironment (TIME) has an enormous impact on carcinogenesis, metastasis and progression (1, 2). Diverse immune cells in TIME have conflicting effects, tumor-antagonizing in some cases and tumor-promoting in others (3). Hence, the heterogeneity of immune components is a pivotal factor affecting the interactions between tumor and TIME (4). Myeloid cells are a major heterogeneous cell population in TIME, including dendritic cells (DC), monocytes/macrophages granulocytes and myeloid-derived suppressor cells (MDSCs) (5). Historically, substantial attention has been focused on their role in tumor angiogenesis, invasion and metastasis (6–8). Recently, increasing evidence revealed that myeloid cells are central compartments of immunosuppressive cells, as a major obstacle to immune therapy (6, 9). For example, programmed death-ligand 1 (PD-L1) expression on myeloid cells hampers T cells differentiation into tumor scavenging effector cells (10) and antagonizes the response to anti-PD-1 therapy (11).

Head and neck squamous cell carcinoma (HNSCC) ranks the sixth most common malignant tumor globally, with increasing incidence and over 300,000 deaths annually (12, 13). HNSCC is remarkably heterogeneous. The heterogeneity of HNSCC in part is derived from molecular changes of the parenchyma (1), but also from the intricate and versatile tumor microenvironment (14, 15). In squamous cell carcinomas, the incidence is lower in immunocompetent patients (16), and low immune infiltration has been shown to associate with a poor prognosis (17). Noteworthy, cancer patients with prospering immune responses are more likely to benefit from immune therapy (18). For patients with recurrent and metastatic cancers, anti-PD-1 immunotherapy has been demonstrated to obtain a durable response and overall survival (OS) benefit (19–21). Although HNSCC exhibits higher immune infiltration than other solid tumors (18, 22, 23), only approximately 15% of patients get benefit from treatment responses (19–21). Thus, novel biomarkers need urgent exploration to evaluate the immune status and predict the prognosis and the immunotherapy response. Previously, myeloid cells have been examined individually, generating piecemeal information about their role in the tumor immune response. Nonetheless, the components of myeloid cells are highly heterogeneous and plastic, which indicates that a valid myeloid signature requires integrative analyses.

During the past decades, unveiled massive data were generated by Gene Expression Omnibus (GEO) and The Cancer Genome Atlas (TCGA) (24, 25), providing unique resources for bioinformatics analysis in tumors. In the present study, we first comprehensively investigated the expression pattern and influence on immune infiltration of myeloid signature genes using the TCGA-HNSCC dataset (26) and GEO dataset (GSE65858) (27). Furthermore, a prognostic signature, termed the myeloid gene score (MGS), was constructed and validated as a prognosis biomarker of HNSCC. Besides, our results also suggested that MGS reflected the immune status, infiltration of immune cells in the tumor and response to immunotherapy.

## Materials and Methods

### HNSCC Datasets Acquisition and Data Processing

The RNA-seq profile data and clinical information of 500 HNSCC and 44 adjacent normal samples (**Table S1**) including 32 (27.27%) oral samples and 12 (72.73%) larynx samples, were downloaded from the TCGA database (https://portal.gdc.cancer.gov/) and the cBio Cancer Genomics portal (cBioPortal, https://www.cbioportal.org/). For the TCGA dataset, a total of 498 HNSCC samples with prognosis, two cases with duplication or missing follow-up removed, were finally chosen and randomly assigned into a training cohort (**Table S2**) and a test cohort (**Table S3**). The GSE65858 dataset from GEO database (https://www.ncbi.nlm.nih.gov/geo/) as an external verification cohort, including microarray and clinical data of 270 HNSCC samples. The TNM stage was determined according to the 7th American Joint Committee on Cancer (AJCC) grading system. Clinical features of HNSCC patients are shown in **Table 1**. We performed data analysis using R (v4.0.2, https://www.r-project.org/), and batch effects between different datasets were controlled with the “ComBat” algorithm (28).

**Table 1:** Clinical parameters of HNSCCs patients in the TCGA and GEO databases. Clinical parameters.

Clinical Pareameters	TCGA training cohort		TCGA test cohort		GEO (GSE65858)	
	n = 249	%	n = 249	%	n = 270	%
**Age**						
<60	118	47.39	101	40.56	153	56.67
≥ 60	131	52.61	148	59.44	117	43.33
**Sex**						
Female	68	27.31	64	25.70	47	17.41
Male	181	72.69	185	74.30	223	82.59
**Subsite**						
larynx	55	22.09	56	22.49		
oral	183	73.49	185	74.30		
Pharynx	11	4.42	8	3.21		
**Histologic grade**						
G1 + 2	180	72.29	178	71.49		
G3 + 4	56	22.49	65	26.10		
GX	12	4.82	4	1.61		
NA	1	0.40	2	0.80		
**T classification**						
T1 + 2	83	33.33	92	36.95	115	42.59
T3 + 4	159	63.86	149	59.84	155	57.41
TX	5	2.01	6	2.41		
NA	2	0.80	2	0.80		
**N classification**						
N0	111	44.58	127	51.00	94	34.81
N+	128	51.41	110	44.18	176	65.19
NX	8	3.21	10	4.02		
NA	2	0.80	2	0.80		
**M classification**						
M0	236	94.78	232	93.17	263	97.41
M1	1	0.40	4	1.61	7	2.59
MX	10	4.02	10	4.02		
NA	2	0.80	3	1.20		
**Stage**						
I + II	46	18.47	67	26.91	55	20.37
III + IV	196	78.71	175	70.28	215	79.63
NA	7	2.81	7	2.81		
HPV infection*						
Positive	34	13.65	35	14.06	73	27.04
Negative	208	83.53	203	81.53	196	72.59
NA	7	2.81	11	4.42	1	0.37
**Vital status**						
Deceased	107	42.97	110	44.18	94	34.81
Living	142	57.03	139	55.82	176	65.19

*Subtypes of HPV infection in clinical information were provided by cBioPortal.

The differentially expressed myeloid signature genes between HNSCC and normal samples were conducted using the “*limma*” R package. The cut-off values were set as |Fold change| >1.5 with FDR <0.05. Then, a heat map and a volcano plot were constructed to show the differentially expressed myeloid signature genes.

### Construction of MGS

First, the candidate prognostic genes were identified by Univariate Cox regression analysis, based on *p <*0.05. Subsequently, a prognostic risk model was constructed by Lasso regression analysis in the TCGA training cohort. Then, the risk score, hereafter termed as the MGS, of each patient was calculated as the following formula:

$$Risk\;score\;(MRS)=\Sigma_{i=1}^{n}Coef{(}_{\mathrm{genei}}*{Exp}_{i},$$

where *n* is the number of candidate prognostic genes, *Coef (gene*_i_ *)* is the coefficient of genes determined by Lasso regression analysis, and *Exp_i_* is the expression value. Ultimately, the HNSCC patients were divided into MGS^low^ and MGS^high^ subgroups based on the median value of MGS in the training cohort.

### Gene Set Enrichment Analysis

Gene set enrichment analysis (GSEA) is a computational method conducted to elucidate biological functions enriched in gene sets between two states (29). In our study, GSEA was performed by GSEA v4.0 (https://www.gsea-msigdb.org/), with C2 (c2.cp.kegg. v7.1.symbols.gmt) curated gene sets from the Molecular Signatures Database (MSigDB) (30). After 1,000 permutations for each analysis, the results with *p <*0.05 and FDR <0.25 were considered significantly enriched sets.

### Immune Score and Immune Cell Infiltration Analyses

The immune score and stromal score of each sample were calculated in the TCGA dataset using ESTIMATE (Estimation of Stromal and Immune cells in Malignant Tumor tissues using Expression data) algorithm *via* the “*estimate*” R package (31). Additionally, the composition fractions of 22 tumor-infiltrating immune cell types in each HNSCC sample were yielded using the CIBERSORT (cell type identification by estimating relative subsets of RNA transcripts) algorithm (32). The samples were adopted according to *p <*0.05 with 1,000 permutations.

### Immunotherapy Datasets

Due to the lack of a database of immunotherapy in HNSCC, metastatic urothelial cancer (mUC) and skin melanoma (SKCM) receiving immunotherapy were adopted and analyzed to evaluate the therapeutic predictive value of MGS. The data package of the mUC dataset was downloaded from http://research-pub.gene.com/IMvigor210CoreBiologies, and preprocessed data with “*IMvigor210CoreBiologies*” R package (33). The gene expression and clinical data of the SKCM dataset were obtained from the cBio Cancer Genomics portal (https://www.cbioportal.org/). A total of 298 mUC and 80 SKCM patients with immunotherapy were analyzed to evaluate the MGS.

### Statistical Analysis

All statistical analyses were carried out using R (v4.0.2). Student’s t-test and one-way ANOVA were performed for comparisons between subgroups. Benjamini–Hochberg multiple testing correction method was used to correct *p*-values for controlling the false discovery rate (FDR). Survival curves were generated utilizing the Kaplan–Meier method, compared with the log-rank test. Univariate and multivariate analyses were performed with Cox regression to determine the independent prognostic value of the variables. The diagnostic efficiency of the MGS was estimated using ROC curve analysis with overall survival (OS) data based on the endpoint death. The correlation analysis of the variables was performed using the Pearson correlation test. *P <*0.05 indicates statistically significant differences.

## Results

### Identification of Differentially Expressed Myeloid Signature Genes

**Figure 1:**
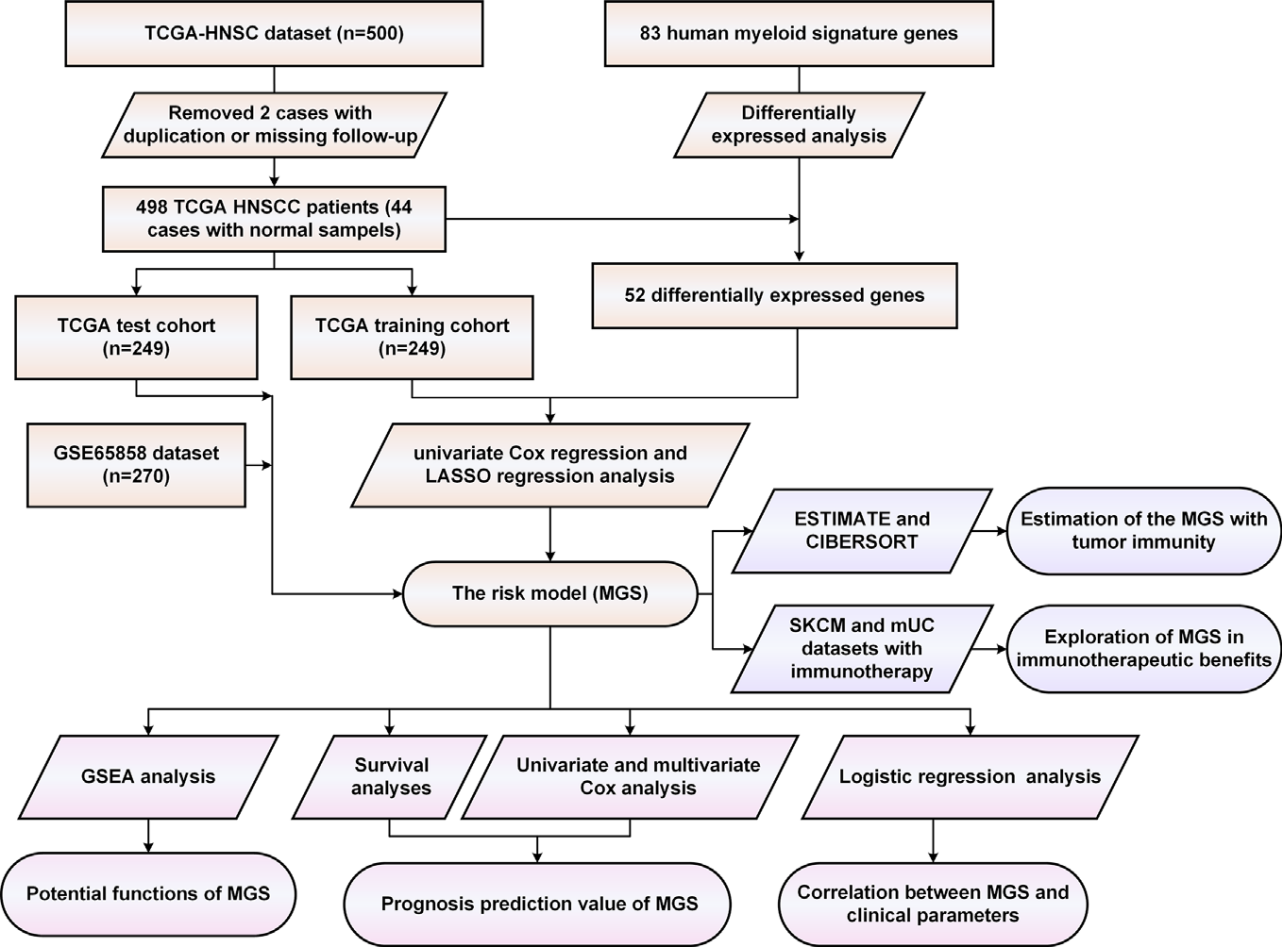
The schematic workflow of the study.

**Figure 2:**
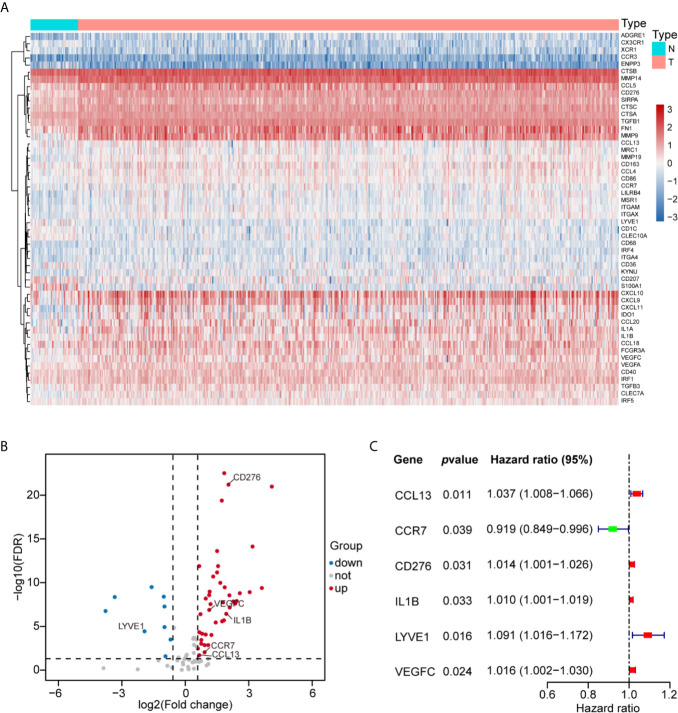
Construction of the MGS with six prognostic myeloid signature genes in HNSCC. **(A)** Fifty-two differentially expressed myeloid signature genes between normal tissue (N) and tumor tissue (T) are shown in the heat map. **(B)** Forty-three upregulated and nine downregulated myeloid signature genes are displayed by a volcano plot (|Fold change| >1.5 and FDR <0.05). **(C)** The six risk myeloid signature genes in the risk model (MGS) are demonstrated by a forest plot.

Initially, we evaluated the expression of 83 human myeloid signature genes (**Table S4**) in 498 HNSCC and 44 adjacent normal tissues. A total of 52 differentially expressed myeloid signature genes were identified (see the schematic workflow in **Figure 1**), including 43 upregulated and nine downregulated genes. The differentially expressed genes are shown in **Table S5** and are displayed with a heat map and a volcano plot (**Figures 2A, B**).

### Construction and Validation of the Prognostic MGS

Then, we evaluated 52 differentially expressed myeloid signature genes in the TCGA training cohort using univariate Cox regression to screen the differentially expressed genes with the prognostic potential to construct a risk model based on the myeloid contexture. Consequently, six prognosis-related myeloid signature genes were identified (**Figure 2C**), which were used to establish a prognostic risk score (MGS) by LASSO regression analysis (**Figure S1**). Detailed information and coefficient of the 6 genes are shown in **Table 2**. MGS of each sample was calculated with the following equation: *Risk score (MGS)* = *CCL13* ∗ *0.0380* + *CCR7* ∗ *(−0.1049)* + *CD276* ∗ *0.0073* + *IL1B* ∗ *0.0034* + *LYVE1* ∗ *0.0673* + *VEGFC* ∗ *0.0020*. According to the median value of MGS (0.246), HNSCC patients in the TCGA training and test cohorts were classified into MGS^low^ and MGS^high^ subgroups.

**Table 2:** List of the six Myeloid signature genes of the MGS in HNSCC.

ENSG ID	Symbol	Location	Expression status	Coefficient
ENSG00000181374	CCL13	Chromosome 17	Upregulated	0.0380
ENSG00000126353	CCR7	Chromosome 17	Upregulated	−0.1049
ENSG00000103855	CD276	Chromosome 15	Upregulated	0.0073
ENSG00000125538	IL1B	Chromosome 2	Upregulated	0.0034
ENSG00000133800	LYVE1	Chromosome 7	Downregulated	0.0673
ENSG00000150630	VEGFC	Chromosome 4	Upregulated	0.0020

**Figure 3:**
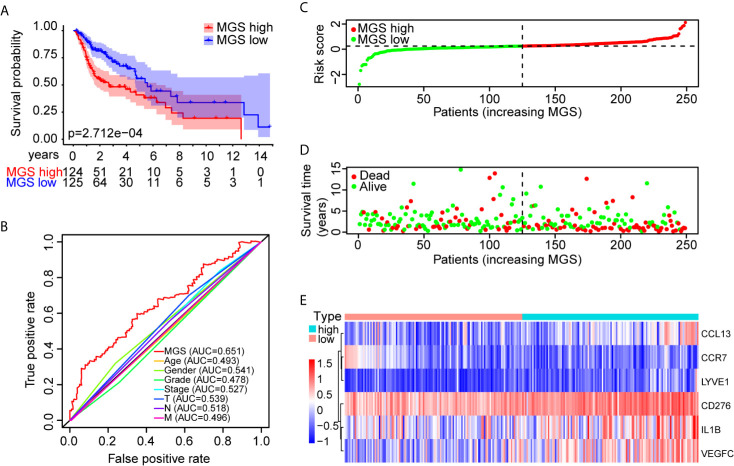
Prognostic performance identification of the MGS in the training cohort. **(A)** Kaplan–Meier survival curve with OS between the patients of MGS^low^ and MGS^high^ subgroups in the TCGA training cohort. **(B)** The discriminatory power of the MGS and other clinical factors shown by the ROC curve of OS in the training cohort. **(C)** The distribution of the MGS^low^ and MGS^high^ HNSCC patients demonstrated by the risk plot. **(D)** The survival state of HNSCC patients displayed by the scatter plot. **(E)** The expression pattern of the six risk genes in the training cohort.

Furthermore, OS analysis was performed separately in the TCGA training and test cohorts to evaluate the prognostic value of MGS. In the training cohort, the prognosis of patients in the MGS^high^ subgroup was markedly worse than patients in the MGS^low^ subgroup (*p <*0.001, **Figure 3A**). The ROC curve analysis indicated that the MGS had a higher ability than other clinical parameters (**Figure 3B**). The MGS and OS status of patients are depicted in dot plots (**Figures 3C, D**). The expression patterns of risk genes in the MGS^low^ and MGS^high^ subgroups presented with a heat map (**Figure 3E**), which revealed that high levels of *CCL13*, *CD276*, *IL1B*, *LYVE1* and *VEGFC* acted as risk factors with high MGS, while high expression levels of *CCR7* acted as a protective factor with low MGS. Similarly, in the TCGA test cohort and TCGA combining set, the prognoses were also different between the MGS^low^ and MGS^high^ subgroups, and the ROC curve analysis also showed that the MGS has a higher ability than other clinical parameters (**Figures S2A, B**). The relationship between the expression patterns of the 6 genes and MGS was in line with the training cohort (**Figures S2C, D**).

To further validate the prognostic performance of MGS, a GEO data cohort (GSE65858) was adopted as an independent external data cohort. According to the same risk model, patients in the GEO test cohort were also segregated into MGS^low^ and MGS^high^ subgroups. As anticipated, prognoses were totally different in these two subgroups (**Figure S3A**), and MGS also had a higher predictive value than other clinical parameters, except for T stages (**Figure S3B**). The MGS and OS statuses are depicted using dot plots (**Figures S3C, D**). The expression profile of the 6 genes was also consistent with the training cohort (**Figure S3E**). Hence, the above results indicated that MGS was robust in multiple validation data cohorts.

### Evaluation of the MGS and the Clinical Parameters

**Figure 4:**
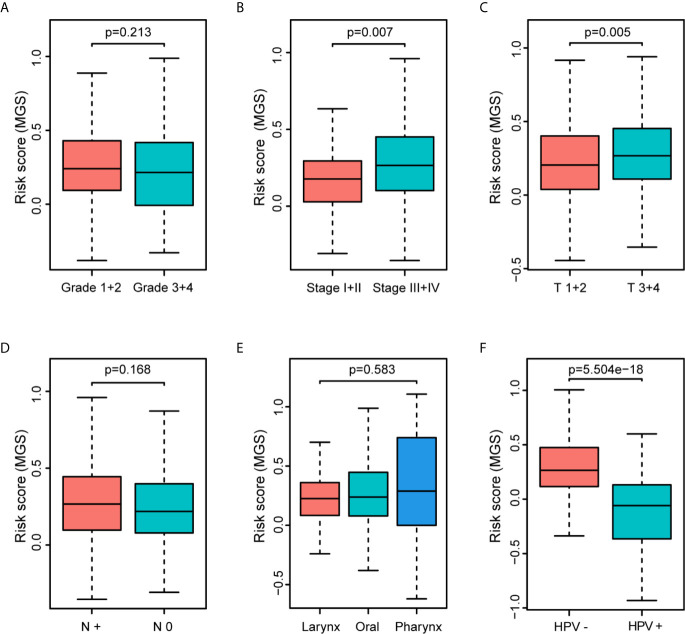
Association of the MGS with clinical parameters of HNSCC patients. Distribution of the MGS stratified by pathologic grade **(A)**, clinical stage **(B)**, T classification **(C)**, N classification **(D)**, subsites **(E)** and HPV infection **(F)** in the TCGA data cohort.

The clinical parameter analysis was performed between the MGS^low^ and MGS^high^ subgroups (**Figures 4A–F**), and the results showed that the MGS of HNSCC patients with Stage III + IV, T3 + 4 and HPV− tumors were higher than those with Stage I + II, T1 + 2 and HPV+ tumors, respectively (*p <*0.001, *p <*0.01 and *p <*0.0001, respectively). Nevertheless, the MGS between the subgroups of Grade, N classification and subsite were not statistically distinct (*P* = 0.213, *P* = 0.168 and *P* = 0.583, respectively). Furthermore, logistic regression was conducted to analyze the association between the MGS and other clinical parameters in the TCGA data cohort (**Table 3**), which demonstrated that MGS was tightly associated with the stage (*p <*0.05), T classification (*p <*0.01) and HPV infection (*p <*0.0001). The results indicated that MGS was closely related to the progression of HNSCC. Univariate and multivariate analysis of OS was performed with the clinical parameters listed in **Table 4**. The results showed that MGS was a potential prognostic factor for HNSCC patients.

**Table 3:** Association between the clinical factors and the MGS in HNSCC patients of TCGA data cohort using logistic regression.

Clinical parameters	Total (N)	Odds ratio in the risk score	*p*-Value
Age (≥60 vs. <60)	498	1.158 (0.813–1.651)	0.417
Sex	498	1.132 (0.760–1.688)	0.543
Subsite	498	1.143 (0.747–1.755)	0.539
Grade (G1 + 2 vs. G3 + 4)	478	0.819 (0.541–1.238)	0.344
Stage (I + II vs. III + IV)	430	1.687 (1.065–2.694)	**0.027**
T classification (T1 + 2 vs. T3 + 4)	442	1.699 (1.169–2.477)	**0.006**
N classification (N0 vs. N+)	404	1.052 (0.734–1.507)	0.783
HPV (Negative vs. Positive)	480	0.097 (0.04–0.203)	**0.000**

**Table 4:** Univariate and multivariate Cox regression of overall survival and clinical parameters in TCGA HNSCCs patients.

Clinical parameters	HR (95% CI)	*p*-value	HR (95% CI)	*p-*value
Age	1.343 (0.979–1.844)	0.068		
Sex	0.804 (0.579–1.116)	0.193		
Subsite	1.009 (0.855–1.191)	0.916		
Grade	0.838 (0.586–1.197)	0.331		
T classification	1.202 (0.864–1.671)	0.274	0.938 (0.519–1.696)	0.833
N classification	1.060 (0.779–1.442)	0.712		
Stage	1.163 (0.810–1.671)	0.414	1.335 (0.693–2.571)	0.388
HPV	0.798 (0.520–1.224)	0.301	0.561 (0.298–1.057)	0.074
MGS	1.696 (1.242–2.315)	**0.001**	1.531 (1.094–2.143)	**0.013**

### GSEA of Enriched Pathways in the Subgroups

**Figure 5:**
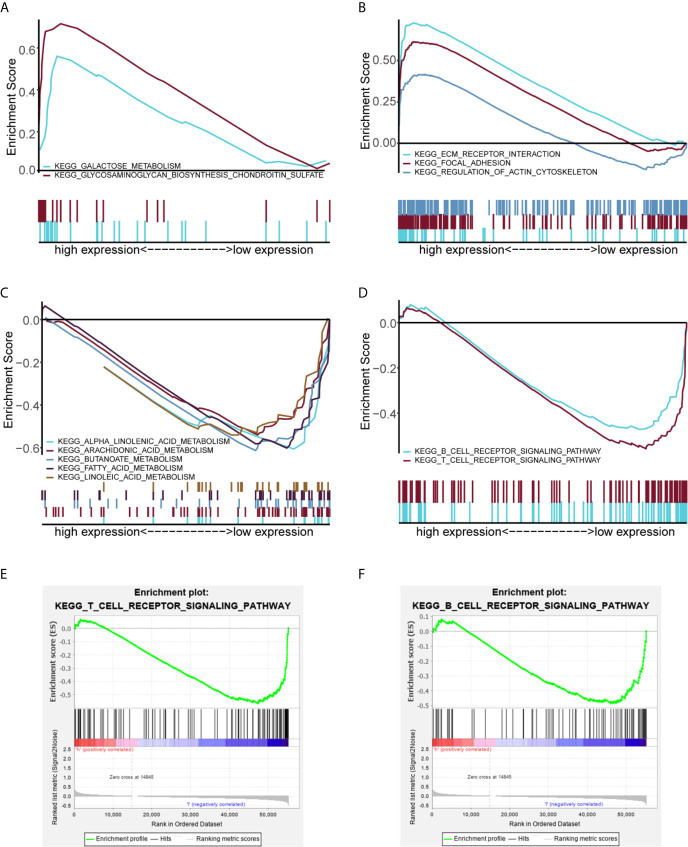
GSEA analysis showing the enriched pathways of the MGS^low^ and MGS^high^ subgroups. Multiple GSEA showing glycometabolism related pathways **(A)** and extracellular matrix (ECM), adhesion and cell cytoskeleton related pathways **(B)** of the MGS^high^ subgroup in the TCGA data cohort. Multiple GSEA showing fatty acid metabolism related pathways **(C)** and immune related pathways **(D)** of the MGS^low^ subgroup. Single GSEA showing the B cell receptor signaling pathway **(E)** and the T cell receptor signaling pathway **(F)** of the MGS^low^ subgroup.

GSEA was performed to identify the potential function of MGS in MGS^low^ and MGS^high^ subgroups in the TCGA data cohort. The top 10 enriched pathways in the MGS^high^ subgroup and 30 enriched pathways in the MGS^low^ subgroup were obtained (**Table S6**), in which signaling pathways with FDR <0.25 and NOM *p <*0.05 were selected (**Table 5**). The results indicated that glycometabolism, extracellular matrix (ECM), adhesion and cell cytoskeleton related pathways were enriched in the MGS^high^ subgroup (**Figures 5A, B**). However, fatty acid metabolism and immune related pathways were enriched in the MGS^low^ subgroup (**Figures 5C, D**). Noteworthy, the B cell and T cell receptor signaling pathway was enriched in the MGS^low^ subgroup (**Figures 5E, F**), which suggested that high MGS may be associated with attenuation of B cell and T cell receptor signaling pathways.

**Table 5:** Gene sets enriched in the MGS^low^ and MGS^high^ subgroups.

MSigDB collection	Name	NES	ES	NOM *p*-val	FDR *q*-val
c2.cp.kegg.v7.1.symbols.gmt	KEGG_ECM_RECEPTOR_INTERACTION	2.139	0.728	0.000	0.004
	KEGG_FOCAL_ADHESION	2.060	0.613	0.000	0.006
	KEGG_GLYCOSAMINOGLYCAN_BIOSYNTHESIS_CHONDROITIN_SULFATE	1.849	0.715	0.002	0.063
	KEGG_GALACTOSE_METABOLISM	1.761	0.560	0.010	0.103
	KEGG_REGULATION_OF_ACTIN_CYTOSKELETON	1.598	0.417	0.024	0.219
	KEGG_ARACHIDONIC_ACID_METABOLISM	−0.554	−1.949	0.000	0.107
	KEGG_T_CELL_RECEPTOR_SIGNALING_PATHWAY	−0.567	−1.932	0.004	0.085
	KEGG_BUTANOATE_METABOLISM	−0.633	−1.928	0.002	0.065
	KEGG_FATTY_ACID_METABOLISM	−0.623	−1.924	0.004	0.054
	KEGG_ALPHA_LINOLENIC_ACID_METABOLISM	−0.664	−1.895	0.000	0.051
	KEGG_LINOLEIC_ACID_METABOLISM	−0.563	−1.671	0.018	0.150
	KEGG_B_CELL_RECEPTOR_SIGNALING_PATHWAY	−0.485	−1.610	0.048	0.159

### Estimation of the MGS With Tumor Immunity

**Figure 6:**
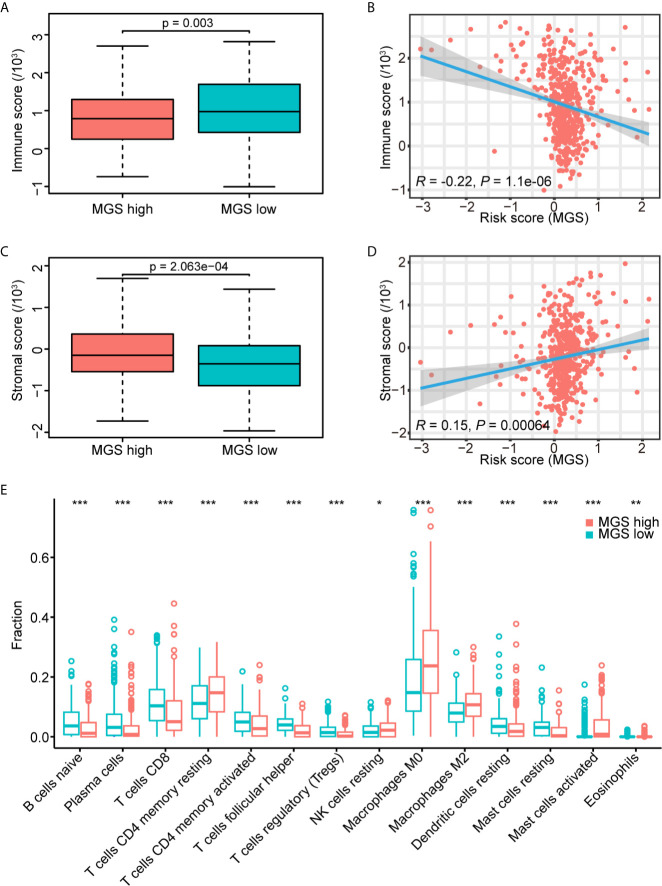
Estimation of the MGS with tumor immunity. **(A)** Distribution of immune scores in the MGS^low^ and MGS^high^ subgroups of the TCGA data cohort. **(B)** Association between the MGS and immune score in HNSCC patients. **(C)** Distribution of stromal scores in the MGS^low^ and MGS^high^ subgroups. **(D)** Association between the MGS and stromal score in HNSCC patients. **(E)** The infiltrating fractions of immune cell types with significant differences in two subgroups. *P < 0.05; **P < 0.01; ***P < 0.001.

Based on the results of GSEA, MGS was associated with tumor immunity. The immune and stromal scores of the TCGA data cohort were estimated to investigate the effect of MGS on tumor immunity using ESTIMATE. The results showed that the immune score of the MGS^low^ subgroup was obviously higher than that of the MGS^high^ subgroup (*p <*0.01, **Figure 6A**), and the MGS was negatively correlated with the immune score (*R* = −0.22, *p <*0.0001, **Figure 6B**). In contrast, patients in the MGS^high^ subgroup had higher stromal scores (*p <*0.001, **Figure 6C**), therefore, MGS was positively associated with the stromal score (*R* = 0.15, *p <*0.001, **Figure 6D**).

Furthermore, the fraction of tumor-infiltrating immune cells in the TCGA data cohort was evaluated using CIBERSORT between MGS^low^ and MGS^high^ subgroups (**Figure 6E**, **Figure S4**). The results showed that the fraction of macrophages (M0) (*p <*0.001), alternative macrophages (M2) (*p <*0.001), activated mast cells (*p <*0.001) and eosinophils (*p <*0.01) in the MGS^high^ subgroup were higher than those in the MGS^low^ subgroup, and the MGS^low^ subgroup represented lower resting dendritic cells (*p <*0.001) and resting mast cells (*p <*0.001). Consistent with the GSEA results as noted in **Figures 5E, F**, the fraction of naïve B cells, CD8^+^ T cells, activated CD4 memory T cells, and follicular helper T cells in the MGS^high^ subgroup were lower than those in the MGS^low^ subgroup (all *p <*0.001), and resting CD4 memory T cells were opposite (*p <*0.001). These results suggested that high MGS was associated with tumor immunosuppression, potentially caused by attenuation of B cell and T cell proliferation and activation. When HPV+ cases were excluded, the results were somewhat skewed, but basically consistent with the analysis of overall cases (**Figure S5**).

### Association of MGS Genes With the T Cell and B Cell Subpopulations

**Figure 7:**
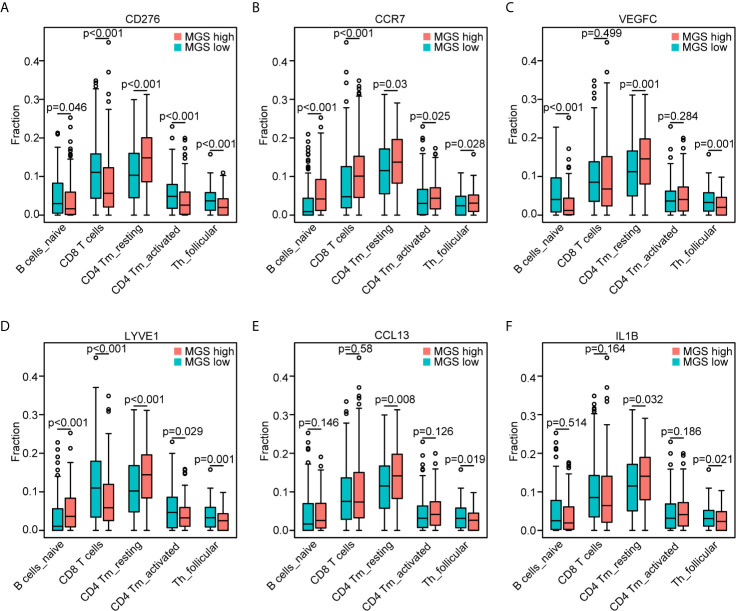
Association of the MGS genes with the T cell and B cell subpopulations. Consistent with**Figure 6E**, the distribution of the five subpopulations of B cells and T cells based on the high and low expression of CD276 **(A)**, CCR7 **(B)**, VEGFC **(C)**, LYVE1 **(D)**, CCL13 **(E)** and IL1B **(F)**, respectively.

According to the associations between MGS and the abovementioned five subpopulations of B cells and T cells, we further explored the potential associations of MGS genes and the five subpopulations of B cells and T cells (**Figures 7A–F**). In line with the expression patterns of the MGS genes, the reduction of naïve B cells was associated with high levels of *CD276* (*p <*0.05), *VEGFC* (*p <*0.001), *LYVE1* (*p <*0.001) and low expression of *CCR7* (*p <*0.001). The attenuation of CD8^+^ T cells was related to a high expression of *CD276* (*p <*0.001), *LYVE1* (*p <*0.001) and low expression of *CCR7* (*p <*0.001). In addition, the decrease of activated CD4 memory T cells was related to the high expression of *CD276* (*p <*0.001), *LYVE1* (*p <*0.05) and low expression of *CCR7* (*p <*0.05). Similarly, the asthenia of follicular helper T cells was linked to high levels of *CD276* (*p <*0.001), *VEGFC* (*p <*0.01), *LYVE1* (*p <*0.001), *CCL13* (*p <*0.05) and *IL1B* (*p <*0.05), while low levels of *CCR7* (*p <*0.05). Moreover, the increase of resting CD4 memory T cells was associated with high expression of *CD276* (*p <*0.001), *VEGFC* (*p <*0.01), *LYVE1* (*p <*0.001), *CCL13* (*p <*0.01) and *IL1B* (*p <*0.05). Thus, MGS genes including CD276, CCR7, VEGFC, LYVE1, CCL13 and IL1B were potentially associated with an immunosuppressive status in patients with HNSCC.

### The Exploration of MGS in Immunotherapeutic Benefits

Emerging immunotherapies for cancer have shown surprising results, but they are only effective in some patients. Hence, we performed subsequent analyses to explore the predictive value of MGS in immunotherapeutic benefits. The mUC and SKCM datasets, which received immunotherapy, were adopted to evaluate the immune score, the expression of PD-L1 and PD1 and response to immunotherapy. Consistently, the immune score of the MGS^low^ subgroup was elevated compared with the MGS^high^ subgroup in the SKCM cohort (*p <*0.0001, **Figure S6A**). Notably, the results showed that the expression of PD-L1 and PD1 in the MGS^high^ subgroup was lower than that in the MGS^low^ subgroup in the SKCM cohort (*p <*0.01 and *p <*0.0001, **Figures S6B, C**). We also found that lower MGS was related to a better response to immunotherapy in the SKCM cohort (**Figure S6D**). Similar results were similarly obtained in the mUC cohort (**Figures S6E–H**). Collectively, the results revealed that MGS was associated with patient response to immunotherapy in the SKCM and mUC cohorts.

## Discussion

The “hot” and “cold” status of tumors are determined by the infiltration of immune cells, and patients with “cold” tumors have a poor prognosis and benefit less from immunotherapy compared with those with “hot” tumors (34, 35). Myeloid cells, crucial components of the TIME, play a critical role in tumor immune evasion and affect treatment outcome (36). Nonetheless, the component heterogeneity and functional variability of myeloid cells bring a great challenge to distinguish their tumor-antagonizing and tumor-promoting effects in tumors (36, 37). So far, the role of the myeloid cells in HNSCC remains ambiguous, which requires an integrative analysis to explore the expression patterns and prognostic significance of myeloid signature genes, especially their association with TIME in HNSCC patients. Here, for the first time, we comprehensively investigated myeloid signature genes and constructed a myeloid signature MGS to reflect the immune infiltration and immune status in patients with HNSCC.

Myeloid cells are important defenders belonging to the innate immune system and crucial in the orchestration of immune responses. Through directly regulating cancer cells and indirectly impinging the cancer stroma, myeloid cells are multifaceted in cancer progression (36–38). Therefore, integrally evaluating the exact roles of myeloid signature genes in cancers could provide valuable information in the prediction of prognosis and determination of treatment strategies. In our MGS model, high levels of *CCL13*, *CD276*, *IL1B*, *LYVE1* and *VEGFC* were risk factors, while a high level of *CCR7* was a protective factor. However, some of the six genes in the heat map by MGS^low^ and MGS^high^ subgroups are not very well differentiated, which may be related to poor discriminations between high and low levels of the genes themselves. *CD276* (B7-H3), a member of the B7 superfamily, was shown to be overexpressed in colorectal cancer, pancreatic cancer, diffuse brain glioma, non-small cell lung cancer and head and neck cancers (39–43), and enhanced cancer progression (44, 45). Accumulating evidence has demonstrated that elevated expression and polymorphisms of *IL1B* acted as a crucial risk factor in various cancers, involving cervical cancer, breast cancer, non-small cell lung cancer (46–48). *LYVE1* (lymphatic vessel endothelial hyaluronan receptor-1) has been identified as a biomarker of lymphangiogenesis and lymph node metastasis in multiple cancers with poor prognoses (49–51). *VEGFC*, as previously shown, is a critical determinant in various signaling pathways catalyzing aggressiveness in multiple cancers (52–55). Our results, consistent with the previous studies, indicated that *CD276*, *IL1B*, *LYVE1* and *VEGFC* are associated with cancer progression. *CCR7* (chemokine receptor 7) can inhibit angiogenesis according to the previous investigation (56, 57), although available studies have shown that it fosters tumor progression (58–60). There are few studies focused on the role of *CCL13* in tumors, and for the first time, we explored it as a risk factor for MGS in patients with HNSCC.

Intriguingly, our GSEA data indicated that the B cell receptor (BCR) and T cell receptor (TCR) signaling pathways were enriched in the MGS^low^ subgroup, which indicated that anti-tumor immune status may be attenuated in the MGS^high^ subgroup (61–63). Consistently, our study revealed that high MGS was associated with an immunosuppressive status, which was reflected by a diminished immune score and decreased infiltration of naïve B cells, CD8^+^ T cells, CD4^+^ memory activated T cells and follicular helper T cells, and elevated infiltration of CD4 memory resting T cells. Meanwhile, the promotion of MGS correlated with the increased infiltration of macrophages (M0), alternative macrophages (M2), activated mast cells and eosinophils. Tumor-associated macrophages (TAMs) composed of multiple subpopulations are generally cataloged as inactivated (M0), classically (M1) and alternatively (M2) activated cells. Indeed, M2 cells can display protumor properties deriving from directly impacting multiple steps in tumorigenesis and development of malignant cells and negatively interacting with other immune cells, involving CD8^+^ T cells (64–66). Similar to M2 cells, mast cells are often identified as tumor-promoting cells (67–69). Previous studies showed that the attenuation of CD276 can elicit suppression of multiple tumors, which depended on CD8^+^ T cells (70, 71). The previous study has investigated the tumor immune infiltration characteristics of HNSCC by multiplex immunohistochemistry. The results revealed that differential immune states according to lymphoid and myeloid cell infiltration were associated with HPV infection and prognosis (72). Consistent with our results, CD8^+^ T cell status correlated with the composition of myeloid populations. Recently, accumulating evidence suggested that immune cell infiltration was closely tied with levels of immune activity in the TIME of HNSCC (15, 73), and immune infiltration could be evaluated using various models constructed by different methods, such as cancer-associated AS events (CASEs) and m^6^A methylation regulators (74, 75). Some of the myeloid genes in our model have been shown to regulate multiple immune cells by previous studies. IL1B, belonging to the IL1 system, can drive senescence-associated secretory phenotypes (SASPs), which may affect immune cell activation and induce neutrophil accumulation (76, 77). In addition, the shed ectodomain of LYVE1 expressed on M2 cells inhibited cancer cell proliferation (78). Furthermore, VEGFC can catalyze TAMs remodeling and CD8^+^ T cells deletion leading to immune escape and immune tolerance (79, 80). Hence, MGS on the basis of 6 prognostic myeloid signature genes may afford a method for evaluating “hot tumors” with myeloid contextures and provide new strategies for the optimization of treatment regimens.

In terms of myeloid cells playing a pivotal role in immunotherapy responses (6, 36), patients who received immunotherapy in mUC and SKCM datasets were obtained to evaluate the difference in immunotherapeutic benefits based on MGS status. Our results showed that the immune score of samples with high MGS were also significantly decreasing compared with those samples with low MGS, and patients in the MGS^high^ subgroup prone to benefit from immunotherapy. These results indicate that the MGS acts as a potential marker to predict immunotherapy response. Therefore, future models utilizing MGS and correlated parameters could facilitate accurately customization of treatment regiments.

However, our investigation has some limitations. Firstly, we performed this study with bioinformatics analysis alone, lacking the validation of solid clinical specimens. The protein expressions of myeloid signature genes in HNSCC at subsites and in different multiplex settings were not verified. Additionally, the research was conducted with a retrospective design rather than a prospective one. The biological basis for MGS differences remains elusive. Furthermore, the heterogeneity of tumor immunity in the subsites of HNSCC deserves further investigation. The immunotherapeutic benefits of MGS were explored with SKCM and mUC datasets, so the evidence for therapeutic response may be insufficient. However, MGS in our study was validated by multiple data cohorts in HNSCC, therefore, our risk model is still reliable and acceptable. Thus, future studies with prospective clinical trials and mechanistic exploration are warranted to further validate the present result and unravel the mechanism modulating the myeloid contexture, however, the evaluation of which will be time-consuming.

In conclusion, our study demonstrated that a novel risk model constructed by six myeloid signature genes could predict prognosis, and assess the myeloid contexture and immune status of HNSCC patients. Moreover, the MGS can facilitate the screening of appropriate candidates for immunotherapy and the development of optimal treatment strategies.

## Data Availability Statement

Publicly available datasets were analyzed in this study. This data can be found here: Expression profile and clinical data of HNSCC datasets can be downloaded from The Cancer Genome Atlas (TCGA) (https://portal.gdc.cancer.gov/) and Gene Expression Omnibus (GEO) database (https://www.ncbi.nlm.nih.gov/geo/). The mUC dataset was downloaded from http://research-pub.gene.com/IMvigor210CoreBiologies. The gene expression and clinical data of SKCM dataset can be obtained from the cBio Cancer Genomics portal (https://www.cbioportal.org/).

## Author Contributions

YL and XZ designed the study and approved the manuscript. ZL contributed to the data analysis and the manuscript writing. HC, DZ, CL, GL, HL, DH, XW, and FZ conducted data interpretations and depicted the figures. All authors contributed to the article and approved the submitted version.

## Funding

This study was supported by the National Natural Science Foundation of China (Nos. 82073009, 81974424, 81874133, 81773243 and 81772903), the National Key Research and Development Project of China (Nos. 2020YFC1316900, 2020YFC1316901), the Natural Science Foundation of Hunan Province (Nos. 2019JJ40481, 2019JJ50944 and 2019JJ50547), the Huxiang Young Talent Project (No. 2018RS3024) and the Fundamental Research Funds for the Central South University (No. 1053320182447).

## Conflict of Interest

The authors declare that the research was conducted in the absence of any commercial or financial relationships that could be construed as a potential conflict of interest.

## Supplementary Material

Supplementary Figure 1 LASSO regression analysis conducted to construct the MGS. **(A)** Expression of 6 risk genes in peritumor and intratumor in HNSCC of TCGA data cohort. **(B)** LASSO algorithms used to identify prognosis-related myeloid signature genes. **(C)** LASSO coefficient values used to construct the MGS in the training cohort. **(D)** Correlation between the 6 risk genes and the MGS. (P < 0.05; Spearman rank correlation).

Supplementary Figure 2 Verification of the MGS in HNSCC patients with the TCGA data set. **(A)** The prognostic performance of the MGS in the TCGA test cohort. (B) The risk plot distribution, survival status, and the expression of six genes in the TCGA test cohort. **(C)** The prognostic performance of the MGS in the TCGA all cohort. **(D)** The risk plot distribution, survival status, and expression of six genes in the TCGA all cohort.

Supplementary Figure 3 Validation of the MGS in HNSCC patients using the GEO data cohort. **(A)** The prognoses in the MGS^low^ and MGS^high^ subgroups of the GEO data cohort. **(B)** The predictive performance of the MGS and other clinical factors shown by the ROC curve in the GEO data cohort. **(C)** The risk score distribution of MGS^low^ and MGS^high^ subgroups. **(D)** Scatter plot showing the survival statuses of HNSCC patients. **(E)** The expression of six genes in the GEO data cohort.

Supplementary Figure 4 Immune cells infiltration in subgroups of the TCGA data cohort. The infiltrating fractions of 22 immune cell types in the MGS^low^ and MGS^high^ subgroups.

Supplementary Figure 5 Estimation of the MGS with immune infiltration in the HNSCC patients excluding the HPV+ cases. The infiltrating fractions of immune cell types with significant differences in the MGS^low^ and MGS^high^ subgroups.

Supplementary Figure 6 The value of the MGS in the prediction of immunotherapeutic benefits. **(A)** Distribution of immune scores in subgroups of the SKCM cohort. The expression distribution of PD-L1 **(B)** and PD1 **(C)** in subgroups of the SKCM cohort. **(D)** Rate of response, stable disease (SD)/progressive disease (PD) and complete response (CR)/partial response (PR), to immunotherapy in subgroups of the SKCM cohort. **(E)** Distribution of immune scores in subgroups of the mUC cohort. The expression distribution of PD-L1 **(F)** and PD1 **(G)** in subgroups of the mUC cohort. **(H)** Rate of response, SD/PD and CR/PR, to immunotherapy in subgroups of the mUC cohort.

Table S1 The list of 44 adjacent normal samples in the TCGA- HNSCC dataset.

Table S2 The HNSCC patients of the TCGA training cohort.

Table S3 The HNSCC patients of the TCGA test cohort.

Table S4 The list of Myeloid signature genes.

Table S5 Fifty-two differentially expressed Myeloid signature genes.

Table S6 The top 10 and 30 enriched pathways in MGS^low^ and MGS^high^ subgroups, respectively.
