# Supplementary figures and images for: Comprehensive Analysis of Myeloid Signature Genes in Head and Neck Squamous Cell Carcinoma to Predict the Prognosis and Immune Infiltration
Source: Front Immunol. 2021 Apr 29;12:659184. doi: 10.3389/fimmu.2021.659184 (PMC8116959; doi:10.3389/fimmu.2021.659184)

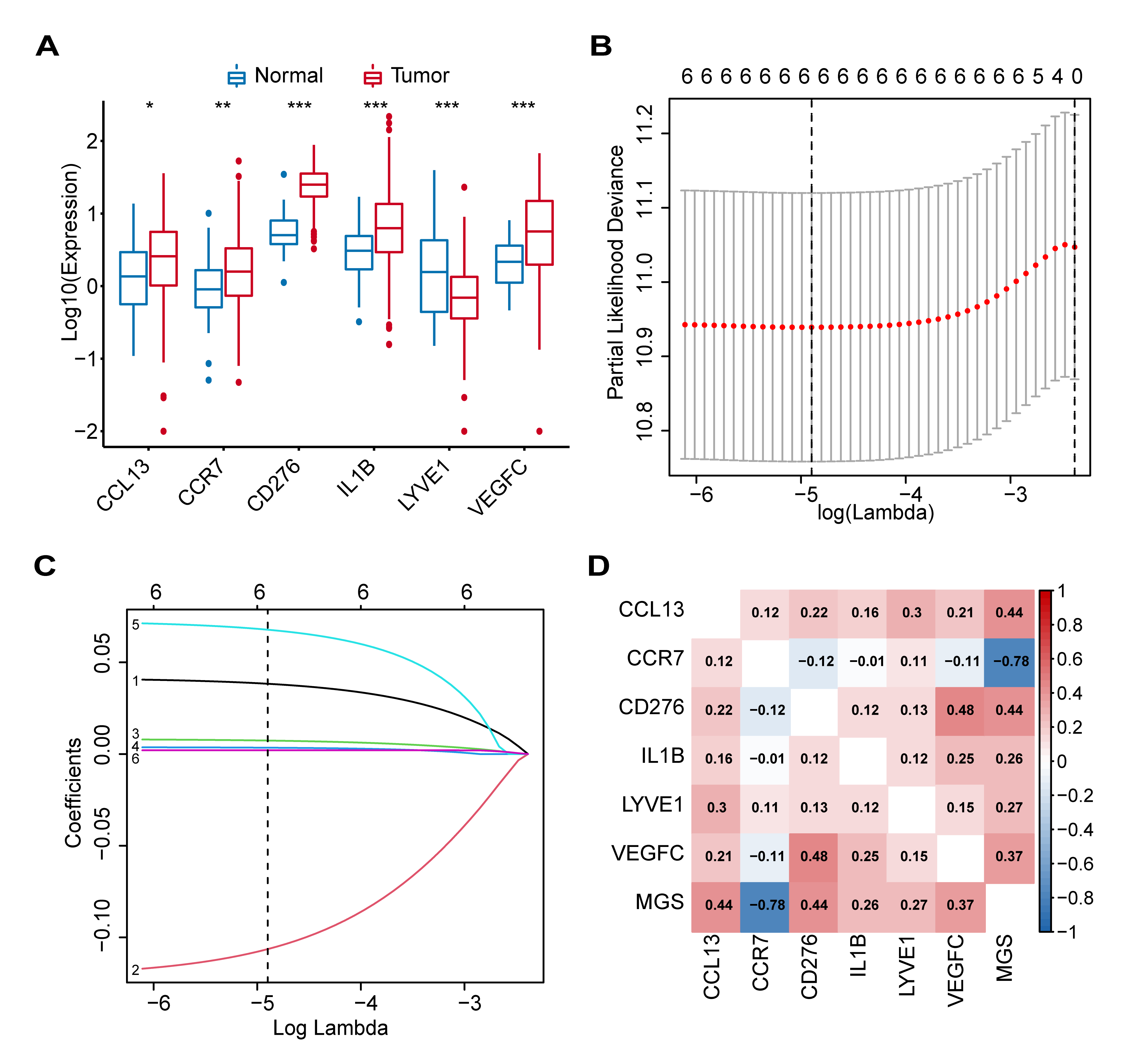

Supplement: Supplementary Figure 1 — LASSO regression analysis conducted to construct the MGS. (A) Expression of 6 risk genes in peritumor and intratumor in HNSCC of TCGA data cohort. (B) LASSO algorithms used to identify prognosis-related myeloid signature genes. (C) LASSO coefficient values used to construct the MGS in the training cohort. (D) Correlation between the 6 risk genes and the MGS. (P < 0.05; Spearman rank correlation). [file DataSheet_1.zip › Supplementary Materials/FS1.LASSO regression analysis+boxplot-01.tif]

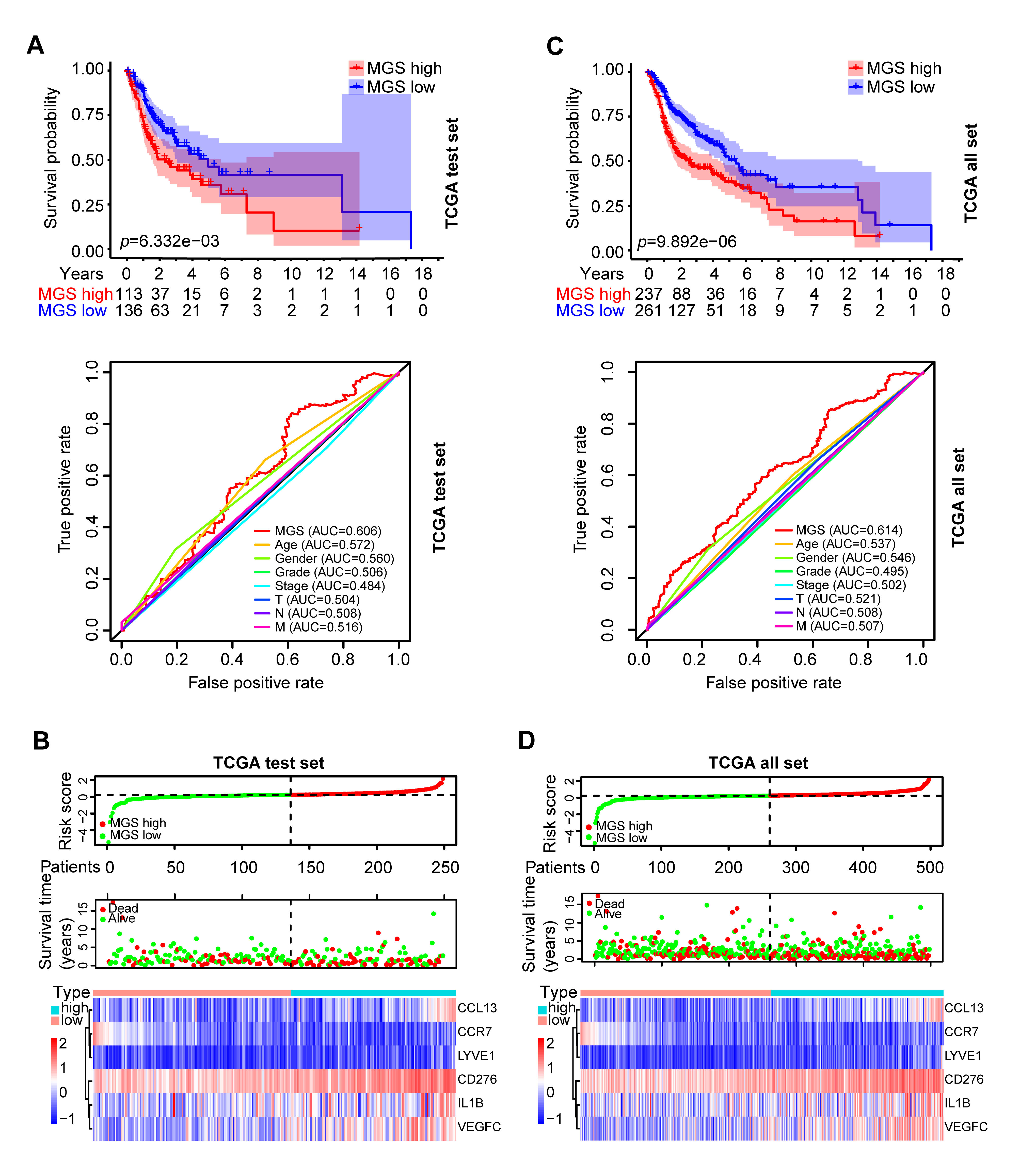

Supplement: Supplementary Figure 1 — LASSO regression analysis conducted to construct the MGS. (A) Expression of 6 risk genes in peritumor and intratumor in HNSCC of TCGA data cohort. (B) LASSO algorithms used to identify prognosis-related myeloid signature genes. (C) LASSO coefficient values used to construct the MGS in the training cohort. (D) Correlation between the 6 risk genes and the MGS. (P < 0.05; Spearman rank correlation). [file DataSheet_1.zip › Supplementary Materials/FS2.the TCGA test and all of 6 genes-01.tif]

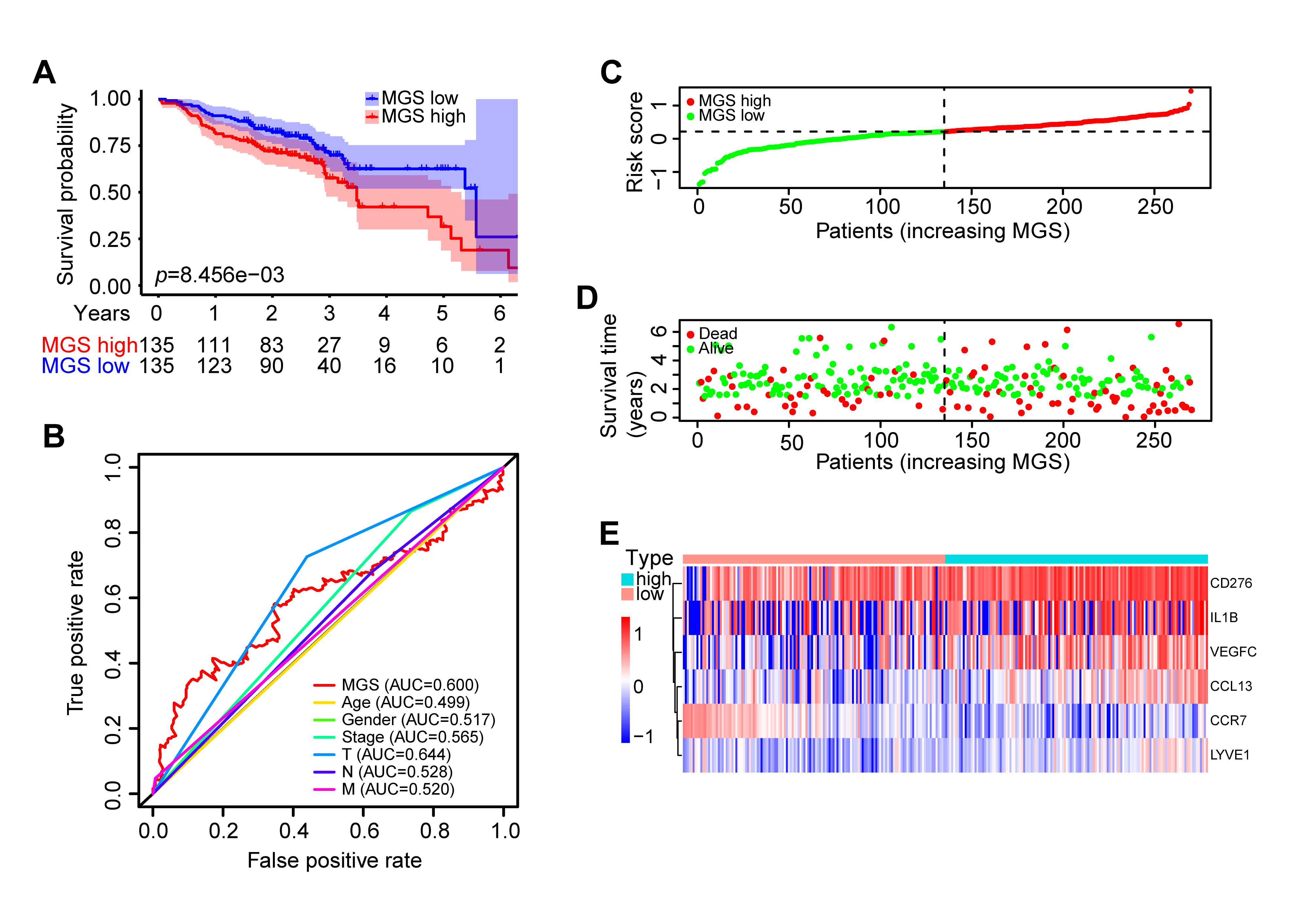

Supplement: Supplementary Figure 1 — LASSO regression analysis conducted to construct the MGS. (A) Expression of 6 risk genes in peritumor and intratumor in HNSCC of TCGA data cohort. (B) LASSO algorithms used to identify prognosis-related myeloid signature genes. (C) LASSO coefficient values used to construct the MGS in the training cohort. (D) Correlation between the 6 risk genes and the MGS. (P < 0.05; Spearman rank correlation). [file DataSheet_1.zip › Supplementary Materials/FS3.the GEO test of 6 genes-01.tif]

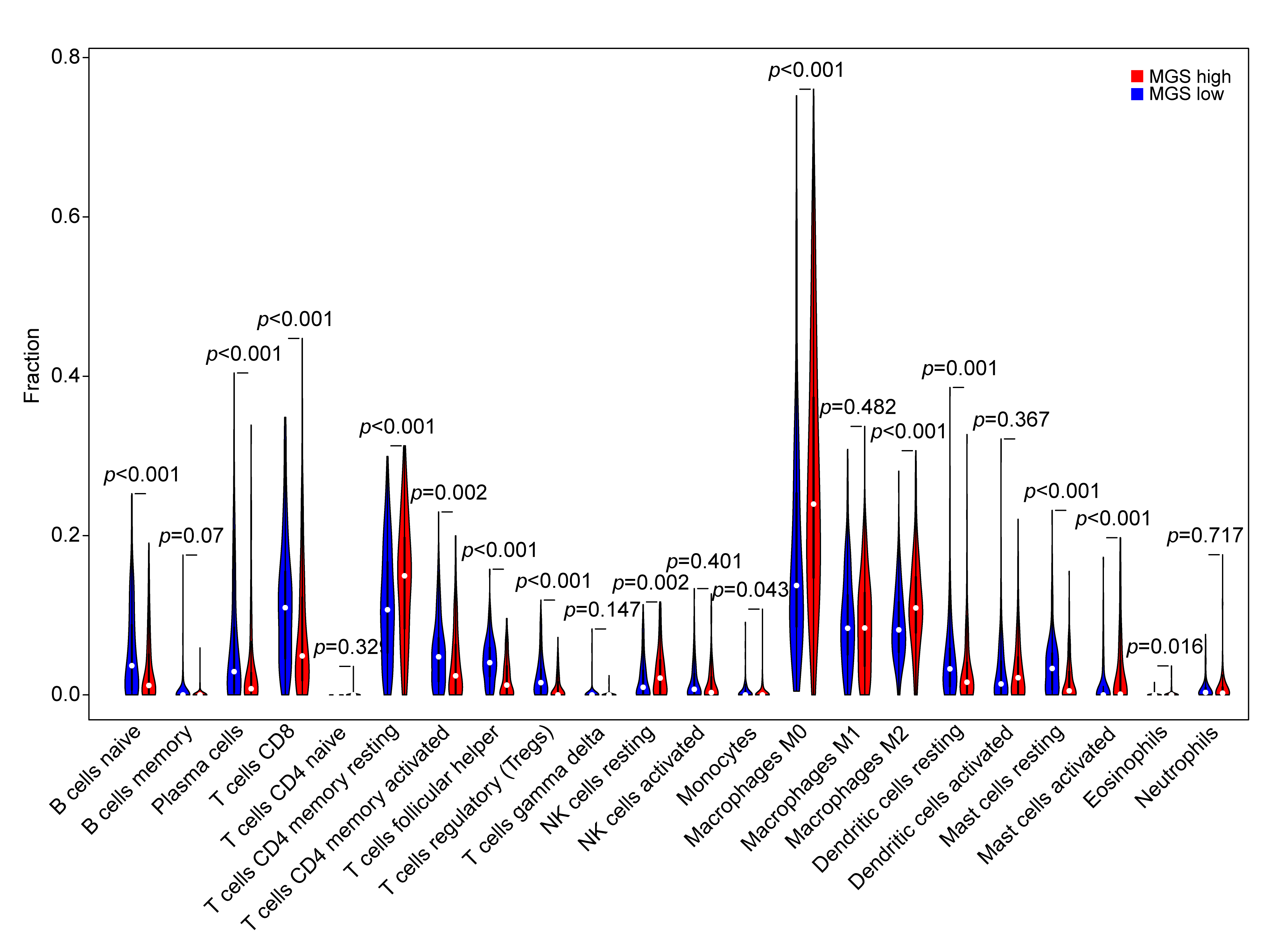

Supplement: Supplementary Figure 1 — LASSO regression analysis conducted to construct the MGS. (A) Expression of 6 risk genes in peritumor and intratumor in HNSCC of TCGA data cohort. (B) LASSO algorithms used to identify prognosis-related myeloid signature genes. (C) LASSO coefficient values used to construct the MGS in the training cohort. (D) Correlation between the 6 risk genes and the MGS. (P < 0.05; Spearman rank correlation). [file DataSheet_1.zip › Supplementary Materials/FS4.immune cells all-01.tif]

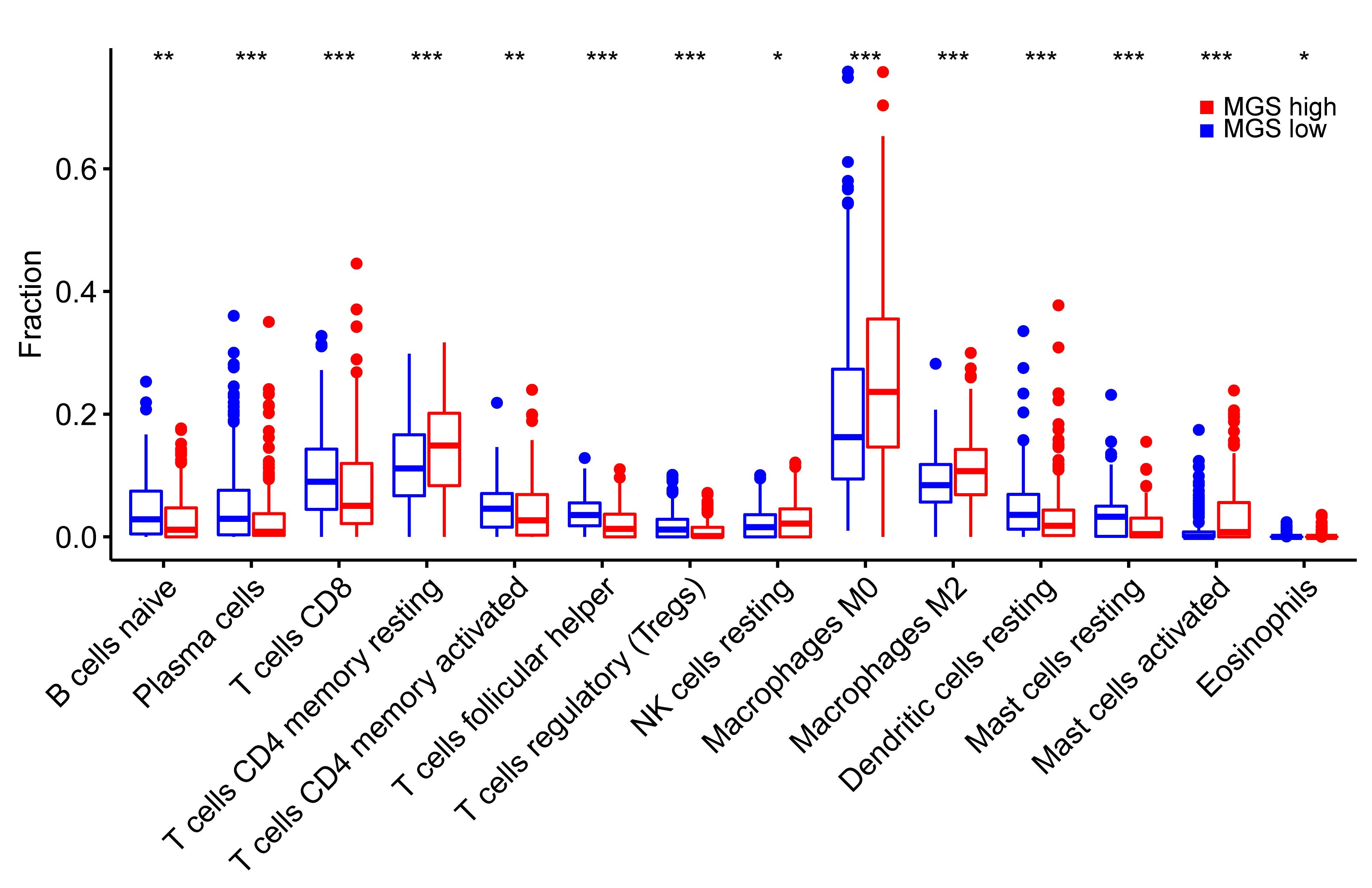

Supplement: Supplementary Figure 1 — LASSO regression analysis conducted to construct the MGS. (A) Expression of 6 risk genes in peritumor and intratumor in HNSCC of TCGA data cohort. (B) LASSO algorithms used to identify prognosis-related myeloid signature genes. (C) LASSO coefficient values used to construct the MGS in the training cohort. (D) Correlation between the 6 risk genes and the MGS. (P < 0.05; Spearman rank correlation). [file DataSheet_1.zip › Supplementary Materials/FS5-immune all -HPV-NA-01.tif]

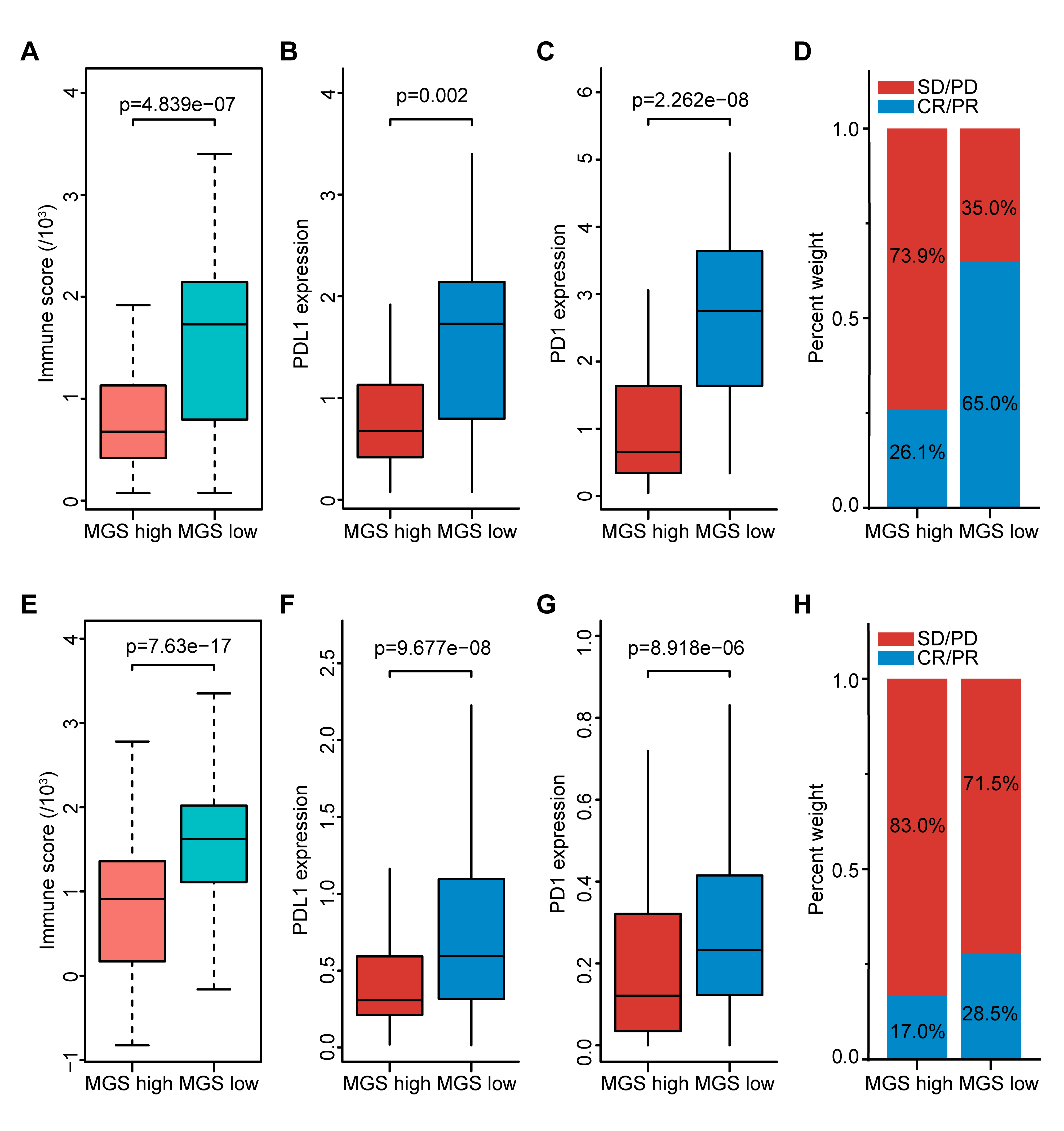

Supplement: Supplementary Figure 1 — LASSO regression analysis conducted to construct the MGS. (A) Expression of 6 risk genes in peritumor and intratumor in HNSCC of TCGA data cohort. (B) LASSO algorithms used to identify prognosis-related myeloid signature genes. (C) LASSO coefficient values used to construct the MGS in the training cohort. (D) Correlation between the 6 risk genes and the MGS. (P < 0.05; Spearman rank correlation). [file DataSheet_1.zip › Supplementary Materials/FS6.immune therapy-01.tif]
